# Large deflection response of restrained steel beams under fire and explosion loads

**DOI:** 10.1186/s40064-016-2509-6

**Published:** 2016-06-17

**Authors:** Feng Xi

**Affiliations:** School of Civil Engineering, Shandong Jianzhu University, Jinan, 250101 People’s Republic of China

**Keywords:** Restrained steel beam, Fire, Blast loads, Computing model, Large deflection, Catenary effect, Dynamic response, Limiting temperature, Pressure–impulse diagram, Numerical study

## Abstract

A numerical study on the response of steel beams to fire and explosion is presented in this paper. First, a unified computing model, which can be used to simulate the behaviour of beams under four loading scenarios that involve fire and explosion or impact, is constructed. The proposed technique allows complete transparency of the influence of the various parameters on the structural behaviour. Second, the effects of load level on critical temperature are analyzed for various static and explosion load ratios. Two cases of explosion and fire loading sequences are compared, it is shown that the critical temperature of the beam subjected to fire followed by an explosion is lower than that of the beam subjected to an explosion followed by fire. The influence of temperature on the p–i diagram is investigated, an iso-damage surface is introduced to distinguish safe and unsafe regions. Third, the limiting temperature criteria are further examined. That is, the first and second limiting temperatures can be determined when the dimensional catenary force is equal to zero or to one, which corresponds to the bending or stretching plastic hinge, respectively.

## Background

After the 9/11 attacks in 2001, the response and failure of structures under the combined loads of impact, explosion, and fire has become a major concern in building design (Maria et al. 2011). The demand for a critical understanding of the response and failure mechanism of main structural members, such as beams and columns that are subjected to multiple extreme loads in steel structures, has increased.

The case of extreme loads including impact and fire can be divided into the following scenarios: (a) blast or impact load, (b) fire load, (c) blast or impact load followed by fire, and (d) blast or impact load during fire.

The load and response process for scenario (a) is short (time is measured in ms). An effective explicit integration technique has been developed to simulate the behavior of structures in current finite element programs (Anonymous 2014). The finite element method (FEM) can predict the dynamic response of structures more accurately than the long-established rigid plastic method because secondary effects such as elasticity, large deformation, strain rate, and so on, are included in calculating the former.

The current analysis for scenario (b) is regarded as a static problem because a fire load is not short-lived (the unit of time is h). The experimental results have shown that the fire resistance of an isolated steel beam with simple supports at its ends differs from that of a beam restrained by surrounding structures. One of the main causes of this difference is the effects of catenary action. Current studies on beams under the effects of fire have also shifted toward this direction (Bradford et al. 2008; Liu et al. 2002; Xi and Luan 2012; Yin and Wang 2005). The fire resistance of restrained steel beams can be analyzed by using various methods; the limiting temperature of the structures can then be determined. However, the traditional concept of limiting temperature, which is defined by the rule that the section bending moment is equal to the plastic limiting moment at a corresponding temperature, is only applied to pure bending beams under the assumption of infinitesimal deflection. Apparently, this definition may be inappropriate for large deflection beams, wherein bending moments and axial forces contribute to yield condition. Catenary action has been demonstrated to improve the fire resistance of restrained beams (Liu et al. 2002). However, the definition of limiting temperature (or failure temperature) remains unclear. Although both limiting temperature criteria were proposed by Xi and Luan (2012), additional examples are needed to check their validity.

In scenario (c), the blast results in fire. The behavior of the structure under fire is near explosion, and the entire course—from blast to fire—can be regarded as a dynamic behavior because it has time and inertial effects. Related studies on this subject have been published (Chen and Liew 2005; Song et al. 2000; Izzuddin et al. 2000; Liew 2008; Liew and Chen 2004). The response of steel frames to a local blast followed by a fire has been investigated. However, such research remains relatively rare even for single steel beams. Simultaneously, developing new methods to address this problem is necessary because of mesh dependency defects in FEM.

The structural response in scenario (d) is more complex than that in scenario (c). In addition, coupling response occurs between thermal and impact effects if structural temperature changes during the course of the blast or impact load and the dynamic response phase. However, even if temperature does not change during the blast or impact response course, and the coupling behavior does not manifest, the dynamic response in the second phase will still be related to material degradation and expansion deformation caused by the elevated temperature during the first phase. Furthermore, although the dynamic properties of a material are also affected by current temperature, experimental data on the relationship between these two factors remain lacking and related literature is scarce. In addition to the study just completed recently (Xi et al. 2014), no papers on the behavior of structures in scenario (d) have been found. However, this scenario is important because temperature seriously affects the pressure–impulse diagram used in the blast-resistance designs of structures (Krauthammer et al. 2008). Hence, considerable related research remains to be done.

For structures under the combined loads of explosion and fire, a unified computing model is required to solve the coupling problem between statics and dynamics. In this manner, the behavior of structures under fire and explosion can be predicted accurately. Hence, this study proposes a unified computing model, in which the statical and dynamic behavior of restrained steel beams under fire, blast, and impact loads can be analyzed more effectively, and the interaction effects between the two types of load can be discussed.

The computing model proposed in this study is established based on the minimum principle of acceleration in the dynamics of elastic–plastic continua at finite deformation (Lee and Ni 1973). Statical analysis is performed by eliminating the inertial effects of the dynamic model, and thus, the catenary effects of steel beams are accurately predicted. Furthermore, by using this dynamic computing model, the response and limiting temperature of steel beams subjected to a blast or impact load followed by fire are discussed. The effects of dead load and explosion intensity ratios on critical temperature are also compared in detail. Finally, this model is developed and can be used to simulate the behavior of steel beams under a fire that follows a blast or impact load. The effects of temperature on the pressure–impulse diagram are discussed, and the existence of an explosion load limit is examined.

## The computing model

To examine the effects of thermal expansion deformation and to simulate the constraint action applied by surrounding structures, a hinged–hinged steel beam with a constant cross section and span *L* is considered. The beam is exposed to fire and subjected to a concentrated load at its mid-span. The basic assumptions made in this model are the same as those in Xi and Luan (2012), Xi et al. (2014).

Under fire and impact loads, the steel beams easily undergo plastic deformation that results from a reduction in their strength and stiffness at elevated temperatures. Thus, the large deflection effects cannot be neglected because catenary action has a significant influence on the behavior of a beam under fire (Yin and Wang 2005).

The minimum principle of acceleration in the dynamics of elastic–plastic continua at finite deformation (Lee and Ni 1973), can be used to derive the governing equations and to solve for a structural member easily and directly. In fact, expressions of a geometric displacement field based on the finite strain are adopted, and thus, large deflection effects are included in motion equations. The principle is perfect to describe the elastic plastic behavior of structures under extreme loads. At present, this principle is used to derive the computing model of steel beams under fire and impact loads.

The minimum acceleration principle is given by:

1 $$J = \int\limits_{{V_{0} }} {S^{ij} \ddot{E}_{ij} dV_{0} + \frac{1}{2}\int\limits_{{V_{0} }} {\rho \ddot{U}^{k} \ddot{U}_{k} dV_{0} - \int\limits_{{A_{T} }} {T^{k} \ddot{U}_{k} dA - \int\limits_{{V_{0} }} {\rho F^{k} \ddot{U}_{k} dV_{0} } } } } ,$$

in which $S^{ij} ,E_{ij} (i,j = 1,2,3)$ are the Kirchhoff stress tensor and Lagrangian strain tensor, respectively; $U_{k} (k = 1,2,3)$ is the component of the displacement vector; $T^{k} ,F^{k}$ are the components of surface force and body force, respectively; $\rho$ is the initial mass density; $A_{T} ,V_{0}$ are the initial surface of the force boundary and the volume of the body, respectively, as defined in Lee and Ni (1973).

Suppose that $u(x,T,t)$ and $w(x,T,t)$ are the axial and transverse displacements of a point at the centroidal axis of the beam, respectively, at temperature *T*. The displacements at any point in the beam are

2 $$\left\{ {\begin{array}{*{20}l} {U_{x} (x,z,T,t) = u - zw_{,x} } \hfill \\ {U_{y} (x,z,T,t) = 0} \hfill \\ {U_{z} (x,z,T,t) = w} \hfill \\ \end{array} } \right.,$$

where $w_{,x} = \partial w/\partial x$. The Green strain and its acceleration can be expressed as

3 $$\left\{ {\begin{array}{*{20}l} {E_{11} = u_{,x} - zw_{,xx} + \frac{1}{2}(u_{,x} - zw_{,xx} )^{2} + \frac{1}{2}(w_{,x} )^{2} } \hfill \\ {\ddot{E}_{11} = \ddot{u}_{,x} - z\ddot{w}_{,xx} + (\ddot{u}_{,x} - z\ddot{w}_{,xx} )(u_{,x} - zw_{,xx} ) + \ddot{w}_{,x} w_{,x} + (\dot{u}_{,x} - z\dot{w}_{,xx} )^{2} + (\dot{w}_{,x} )^{2} } \hfill \\ \end{array} } \right.,$$

where $E_{11}$ is the axial total strain and is noted by $\varepsilon$ below. It includes two parts as follows:

4 $$\varepsilon = \varepsilon^{\sigma } + \varepsilon^{T} ,$$

where $\varepsilon^{\sigma }$ and $\varepsilon^{T} = \alpha (T - 20)$ are the stress-related strain and thermal strain, respectively.

For the case without body force, suppose that $p(t)$ is the load magnitude per unit of axial length. By substituting Eqs. (2) and (3) into Eq. (1), Lee’s functional *J* is presented as follows:

5 $$\begin{aligned} J = \int_{0}^{L} {\left[ {\frac{{m_{0} }}{2}(\ddot{u}^{2} + \ddot{w}^{2} )} \right]^{2} + \frac{{I_{y} }}{2}(\ddot{w}_{,x} )^{2} ]dx + } \int_{0}^{L} {\int_{A} {\sigma [} } (\ddot{u}_{,x} - z\ddot{w}_{,xx} )(1 + u_{,x} - zw_{,xx} ) + \ddot{w}_{,x} \\ w_{,x} + (\dot{u}_{,x} - z\dot{w}_{,xx} )^{2} + (\dot{w}_{,x} )^{2} ]dAdx - p\left[ {w_{,x} \left( {\ddot{u} - \frac{H}{2}\ddot{w}_{,x} } \right) + \left( {1 + u_{,x} - \frac{H}{2}w_{,xx} } \right)\ddot{w}} \right], \\ \end{aligned}$$

where $m_{0} = \rho A$, $I_{y} = \int_{A} {\rho z^{2} dA}$. The axial force, bending moment, and second-order moment of the stress of the cross section are respectively given by:

6 $$N_{x} = \int_{A} \sigma dA, \quad M_{x} = - \int_{A} \sigma zdA, \quad L_{x} = \int_{A} \sigma z^{2} dA.$$

Equations (5) and (6) can be replaced with their discrete form. The discrete form of motion equations for a hinged–hinged beam can be obtained as follows:

7 $$\left\{ {\begin{array}{*{20}l} {\ddot{u}_{i} = \frac{1}{{2m_{0} \Delta x}}[(1 + A_{{i + 1}} )(N_{x} )_{{i + 1}} - (1 + A_{{i - 1}} )(N_{x} )_{{i - 1}} + (M_{x} )_{{i + 1}} B_{{i + 1}} - (M_{x} )_{{i - 1}} B_{{i - 1}} ]}\\ {[1 + \frac{{I_{y} }}{{2m_{0} (\Delta x)^{2} }}]\ddot{w}_{i} - \frac{{I_{y} }}{{4m_{0} (\Delta x)^{2} }}(\ddot{w}_{{i - 2}} + \ddot{w}_{{i + 2}} ) = \frac{1}{{2m_{0} \Delta x}}\{ B_{{i + 1}} (N_{x} )_{{i + 1}} - B_{{i - 1}} (N_{x} )_{{i - 1}} }\\ { - \frac{2}{{\Delta x}}[(1 + A_{{i + 1}} )(M_{x} )_{{i + 1}} - 2(1 + A_{i} )(M_{x} )_{i} + (1 + A_{{i - 1}} )(M_{x} )_{{i - 1}} + C_{{i + 1}} (L_{x} )_{{i + 1}} }\\ { - 2C_{i} (L_{x} )_{i} + C_{{i - 1}} (L_{x} )_{{i - 1}} ]\} + \frac{p}{{m_{0} }}\quad (i = 2, \ldots ,n - 1)}\end{array} } \right.$$

where

8 $$\left\{ {\begin{array}{*{20}l} {A_{1} = \frac{{4u_{2} - u_{3} }}{2\Delta x},} \hfill & {A_{n} = \frac{{ - 4u_{n - 1} + u_{n - 2} }}{2\Delta x},} \hfill & {A_{i} = \frac{{u_{i + 1} - u_{i - 1} }}{2\Delta x}} \hfill \\ {B_{1} = \frac{{4w_{2} - w_{3} }}{2\Delta x},} \hfill & {B_{n} = \frac{{ - 4w_{n - 1} + w_{n - 2} }}{2\Delta x},} \hfill & {B_{i} = \frac{{w_{i + 1} - w_{i - 1} }}{2\Delta x}\quad (i = 2, \ldots ,n - 1)} \hfill \\ {C_{1} = \frac{{ - 5w_{2} + 4w_{3} - w_{4} }}{{(\Delta x)^{2} }},} \hfill & {C_{n} = \frac{{ - 5w_{n - 1} + 4w_{n - 2} - w_{n - 3} }}{{(\Delta x)^{2} }},} \hfill & {C_{i} = \frac{{w_{i + 1} - 2w_{i} + w_{i - 1} }}{{(\Delta x)^{2} }}} \hfill \\ \end{array} } \right..$$

The preceding motion equation can also be applied to the case of concentrated loads but only by using the Dirac function (Yang and Xi 2003). The transverse weight force can be applied on the beam as load $p(t)$.

The discrete form of the stress-related strain at temperature $T$ is

9 $$\varepsilon^{\sigma } (i,j) = A_{i} - j\Delta zC_{i} + \frac{1}{2}(B_{i} )^{2} - \varepsilon^{T} (i,j).$$

The Cowper–Symonds equation (Cowper and Symonds 1957), which is related to strain rate, is adopted for the steel beam, as shown in the following equation, because the material is sensitive to strain rate:

10 $$\sigma^{T} = \sigma_{s}^{T} [1 + ({}^{T}\dot{\varepsilon }_{p}^{\sigma } /D^{T} )]^{{q^{T} }} ,\begin{array}{*{20}c} {} & {} \\ \end{array} \sigma^{T} \ge \sigma_{s}^{T} ,$$

where $\sigma_{s}^{T}$ is the initial yield stress at temperature $T$; ${}^{T}\dot{\varepsilon }_{p}^{\sigma }$ is the rate of plastic strain related to stress at temperature $T$; and $D^{T}$ and $q^{T}$ are the constants related to the strain rate and temperature, respectively. For fire loads, however, the effects of the strain rate can be ignored because it can be regarded as a static course.

**Fig. 1:**
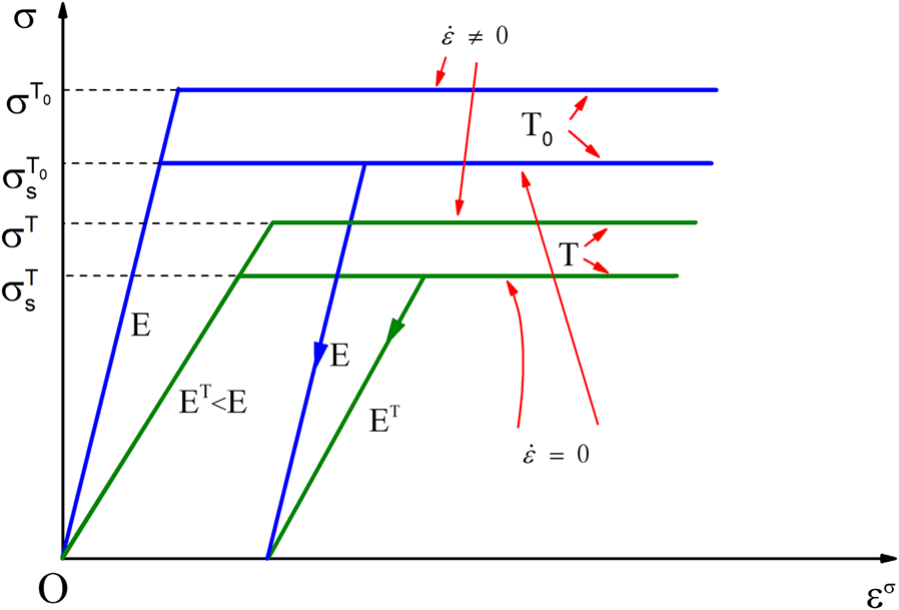
The stress–strain relationship

For the stress–strain relationship, the elastic-perfectly plastic model shown in Fig. 1 is adopted, and the temperature variation in each time step is assumed to have no effect on plastic strain (Franssen 1990).

The discrete forms of relationship between strain increment and stress increment are as follows:

11 $$\left\{ {\begin{array}{*{20}l} {\Delta \sigma^{T} = E^{T} \Delta \varepsilon^{\sigma } ,} \hfill & { - \sigma_{s}^{T} \le \sigma^{T} \le \sigma_{s}^{T} ,or\begin{array}{*{20}c} {} \\ \end{array} unloading} \hfill \\ {\Delta \sigma^{T} = \sigma_{s}^{T} [1 + (\frac{{\Delta \dot{\varepsilon }_{p}^{\sigma } }}{{D^{T} }})]^{{q^{T} }} ,} \hfill & {\left| {\sigma^{T} } \right| > \sigma_{s}^{T} ,and\begin{array}{*{20}c} {} \\ \end{array} loading} \hfill \\ \end{array} ,} \right.$$

where $\Delta \varepsilon^{\sigma }$ and $\Delta \dot{\varepsilon }_{p}^{\sigma }$ are the stress-related strain increment and the rate of stress-related plastic strain increment, respectively, at temperature $T$.

For the relations between $E^{T}$ and $E$, and $\sigma_{s}^{T}$ and $\sigma_{s}$, Eurocode 3 provides the following factors in table form for a common structural steel (Eurocode 2001), as shown in Table 1.

**Table 1:** Retention factors of material strength and stiffness

T (°C)	20	100	200	300	400	500	600	700	800	900	1000	1100
$E_{T} /E$	1.0	1.0	0.9	0.8	0.7	0.6	0.31	0.13	0.09	0.0675	0.045	0.0225
$\sigma_{s}^{T} /\sigma_{s}$	1.0	1.0	1.0	1.0	1.0	0.78	0.47	0.23	0.11	0.06	0.04	0.02

The initial conditions are

12 $$t = 0,\quad \left\{ {\begin{array}{*{20}c} {u_{i} = 0,\dot{u}_{i} = 0} \\ {w_{i} = 0,\dot{w}_{i} = 0} \\ \end{array} ,\quad (i = 1, \ldots ,n)} \right..$$

Equations (8) to (12) comprise the computing model for a hinged–hinged steel beam under fire and transverse distributed loadings, which are expressed by displacements $$u_{i}$$ and $$w_{i}$$$$u_{i,} w_{i}$$ at each node. Several second-order effects such as elastic deformation, strain rate, temperature, rotation inertial, large deflection, and so on are involved in this model. When loads are linearly increased with time, and time is sufficiently long for a loading course, inertial effects can be eliminated and the static computing model can be obtained. Thus, the behavior of a steel beam subjected to a dead load and fire can be analyzed. Note that the governing equations for beams under a concentrated load can be obtained by using Dirac function. In addition, the proposed computing model can express the response problem of steel beams under the separate or combined loads of explosion, fire, and weight loads.

To solve the proposed computing model, the following discrete equations, which are obtained by applying the central finite difference technique to the velocity and acceleration at each node, must be used:

13 $$\left\{ {\begin{array}{*{20}l} {u_{i}^{q + 1} = \ddot{u}_{i}^{q} (\Delta t)^{2} + 2u_{i}^{q} - u_{i}^{q - 1} } \hfill \\ {w_{i}^{q + 1} = \ddot{w}_{i}^{q} (\Delta t)^{2} + 2w_{i}^{q} - w_{i}^{q - 1} } \hfill \\ \end{array} } \right..$$

This computing model can be called the dynamic finite difference method (abbreviated as FD).

## Numerical examples and analysis

Herein several numerical examples are presented. Based on the numerical results, several response features and the limiting temperature of the beam are analyzed, and the interaction effects of structural behavior under two types of loads are discussed.

**Fig. 2:**
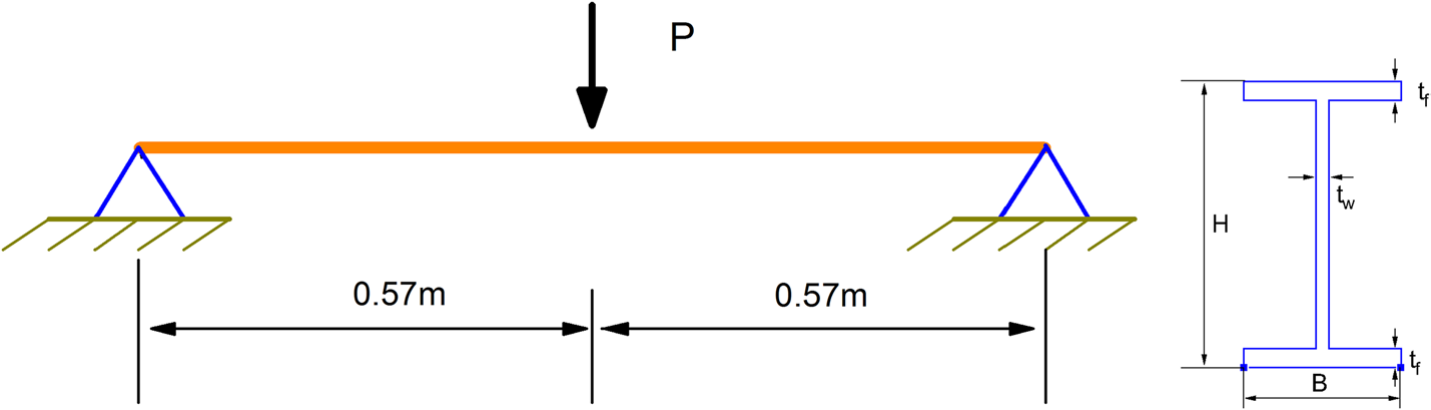
Geometric configuration of a beam with an H cross section

For example, the response of the steel beam with an H cross section shown in Fig. 2 is discussed. Several parameters of the structure, materials, and loads are given as follows.

Geometric dimensions of the beam with an H cross section:

- Span of beam: *L* = 1.14 m
- Height of cross section: *H* = 0.08 m
- Width of flange: *B* = 0.046 m
- Thickness of flange: *t*_*f*_ = 0.0052 m
- Thickness of web: *t*_*w*_ = 0.0038 m
- Material constants of steel at normal temperature:
- Density and Thermal expansion coefficient: $$\rho = 7850$$ kg/m^3^, $$\alpha = 1.4 \times 10^{ - 5} /$$$${}^{\circ}{\text{C}}$$
- Young’s modulus, yield stress and Poisson ratio: $E = 205$ GPa, $\sigma_{s} = 399$ MPa, $\nu = 0.3$
- Strain rate constants: *D* = 40.4 s^−1^, *q* *=* 5

The load ratio is defined by $\eta =$*P/P*_*c*_, in which *P*_*c*_ = *4M*_*P*_*/L*, *M*_*P*_ is the plastic limiting bend moment.

### Steel Beam under elevated temperatures

The deformation behavior of a beam under fire may be seriously affected by the restraining action of the surrounding structures because of thermal expansion. As a reduced beam model in practice, a hinged–hinged beam has been demonstrated to have the best fire-resistant capability (Liu et al. 2002).

#### Two traditional plastic hinges and two concepts of limiting temperature

As is known to all, the effects of thermal expansion and end axial constraints on a hinged–hinged beam at elevated temperatures will result in catenary action. Accordingly, large deflection effects have to been considered and the fire-resistant capability of the beam can be improved. However, in the large deflection case, axial force is also included in the section along with bending moment. The influence of the interaction between these factors on the yield of the material must be considered. Thus, determining the limiting temperatures in such cases is the issue that will be discussed during this phase.

**Fig. 3:**
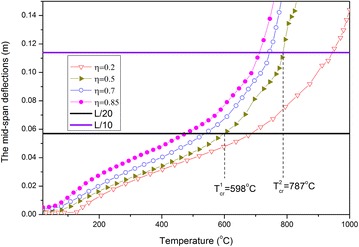
Curves of the mid-span deflection against temperature under different load ratios

Corresponding to several load ratio situations, the curves of the mid-span deflection against temperature are presented in Fig. 3. With regard to the increase in temperature, the deflections do not rapidly change but gradually increase. Thus, determining the failure limiting temperature appears difficult because the axial constraints at the ends of the beam do not only restrain the axial displacements at the ends, but also cause the beam to produce an axial force on the section. Moreover, the early axial contraction force can be changed into a stretch force as temperature increases. Then, the stretching axial force and bending moment jointly resist the loads, and slow down the increase in beam deflections. The aforementioned phenomenon is called the catenary action effect (Yin and Wang 2005).

For a fully fixed steel beam under fire, two criteria to determine the limiting temperature are proposed by Xi and Luan (2012) as follows:

Criterion 1: The first limiting temperature, *T*_*lim*_^1^, can be defined when the dimensionless catenary force is zero or the dimensionless bending moment is one, which correspond to the traditional bending plastic hinge.

Criterion 2: The second limiting temperature, *T*_*lim*_^2^, can be defined when the dimensionless catenary force is one or the dimensionless moment is zero, which correspond to the traditional stretching plastic hinge.

Then, the adaptability of the aforementioned criteria to a hinged–hinged beam is examined.

**Fig. 4:**
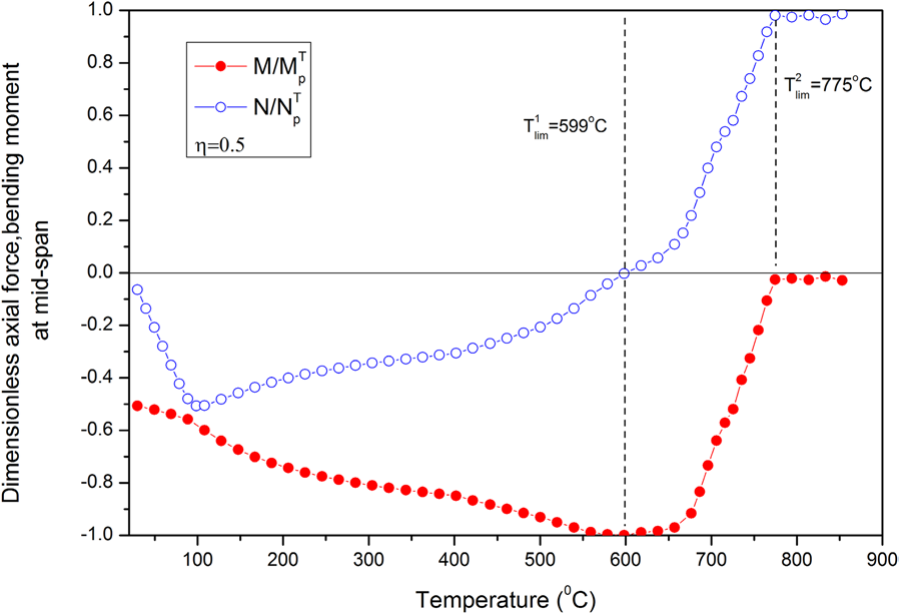
Curves of the dimensionless axial force and bending moment against temperature

The curves of the dimensionless bending moment *M*_*x*_^*T*^*/M*_*P*_^*T*^ and axial force *N*_*x*_^*T*^*/N*_*P*_^*T*^ against temperature are presented in Fig. 4, where *M*_*x*_^*T*^ and *N*_*x*_^*T*^ are the bending moment and axial force of the mid-section at temperature *T*, respectively, whereas *M*_*P*_^*T*^ and *N*_*P*_^*T*^ are the fully bending moment and the full axial force of the corresponding temperature, respectively. The bending moment increases as temperature is raised from 20 °C until the moment when a maximum value of one is reached. However, as temperature rises, the axial force initially increases and then decreases because of the combined effects of thermal expansion and large deflection. The dimensionless axial force is also equal to zero when the bending moment is increased to one. Thus, a traditional bending plastic hinge is formed on the mid-section of the beam. The corresponding temperature, which should be defined as the failure limiting temperature, is called the first limiting temperature *T*_*lim*_^1^. However, as temperature continues to increase, this plastic hinge will not last. Moreover, an increased deflection results in axial tension forces or catenary actions, which cause the steel beam to retain its carrying capacity. Obviously, when temperature exceeds *T*_*lim*_^1^, the moment decreases and the axial force increases as temperature rises. This phenomenon demonstrates that the deformation mode of the steel beam is changed from a mainly bending flexure into a catenary state dominated by the stretching force after the bending plastic hinge disappears. Along with temperature increases, the dimensionless axial force also increases and achieves the maximum value of one, whereas the moment is zero. Thus, a traditional stretching plastic hinge is formed on the section, and the steel beam exhibits full plastic chord status. This stretching plastic hinge will keep up with continuous temperature increases until the steel beam breaks and completely loses its carrying capacity. Thus, at the start of this phase, another failure limiting temperature can be defined and called as the second limiting temperature *T*_*lim*_^2^.

#### Comparison between limiting temperature and critical temperature

The concept of critical temperature in the current code is defined as the state when the maximum deflection of the beam achieves the rated value (such as span/20 or span/10) (Eurocode 2001). The critical temperatures correspond to span/20 and span/10, which are denoted by $T_{cr}^{1}$ and $T_{cr}^{2}$, respectively. Then, for each case of load ratio $\eta$ = 0.2, 0.5, 0.7, 0.85, the corresponding critical temperature can be easily determined by using the curve of the mid-span deflection against the temperature shown in Fig. 3. For example, for $\eta = 0.5$, $T_{cr}^{1}$ = 598 °C, $T_{cr}^{2} =$ 787 °C; whereas, from Fig. 4, both limiting temperatures are $T_{\lim }^{1} = 599$ °C, $T_{\lim }^{2} = 755$ °C. Obviously, $T_{cr}^{1}$ is close to $T_{\lim }^{1}$ but $T_{cr}^{2}$ is slightly higher than $T_{\lim }^{2}$.

**Fig. 5:**
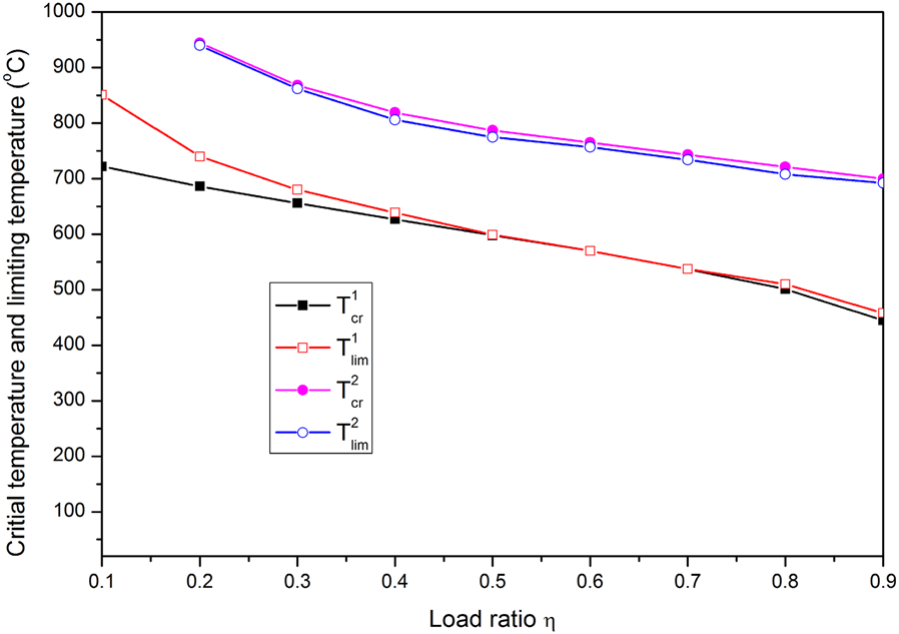
Curves of the limiting temperature and critical temperature against load ratio

According to the aforementioned criteria for limiting temperature and critical temperature, the corresponding temperature against the load ratio can be presented, as shown in Fig. 5. Thus, an intuitive comparison can be made. Again, $T_{cr}^{1}$ and $T_{cr}^{2}$ are close to $T_{\lim }^{1}$ and $T_{\lim }^{2}$, respectively. For the cases with a low load ratio ($\eta < 0.4$), $T_{cr}^{1}$ is slightly lower than $T_{\lim }^{1}$. However, $T_{\lim }^{1}$ is slightly higher than $T_{\lim }^{2}$ for all load ratios.

Based on the preceding numerical results, a suggestion for a fire-resistant design for a hinged–hinged steel beam can be provided. If a large deformation is permitted and the influence of the reaction force at the ends of the surrounding structures can be ignored, then the criterion that the maximum deflection is equal to span/10 [as indicated in Yin and Wang (2005), this displacement is close to the recorded data of the Cardington test] can be used to determine critical temperature $T_{cr}^{2}$. However, if the influence of the reaction force at the ends of the surrounding structures should be considered, then determining critical temperature $T_{cr}^{1}$ by using the criterion that the maximum deflection is equal to span/20 is appropriate. This conclusion corresponds with the case of the fixed–fixed beam discussed in Xi and Luan (2012).

It should be stressed that some results obtained herein are quite already known in the fire community, this indicates that the minimum principle of acceleration can be used to effectively analysis the large deflection static behavior of structures subjected to fire loading. Compared with the method of Newton–Raphson commonly used in the response analysis of structures under fire, the proposed method is more straightforward and simple, and the iterative computation is not need.

### Steel beam subjected to explosion followed by fire

The beam discussed earlier is then subjected to the following loads in succession.

① First, a transverse dead load *P* is applied at the mid-span, $P = \eta P_{c}$.
② Next, an explosion load, which is a rectangular pulse with intensity $P_{d}$ and duration time *t*_0_ = 0.25 s, is applied at the mid-span. The Explosion Load Ratio (ELR in short) is $\eta_{e} = P_{d} /P_{c}$.
③ Fire is applied. Temperature is assumed to be distributed uniformly along each direction of the beam.

Undoubtedly, the response of the steel beam under these combined loads is complicated. At present, the influence of blast loads on failure limiting temperature is examined in particular.

**Fig. 6:**
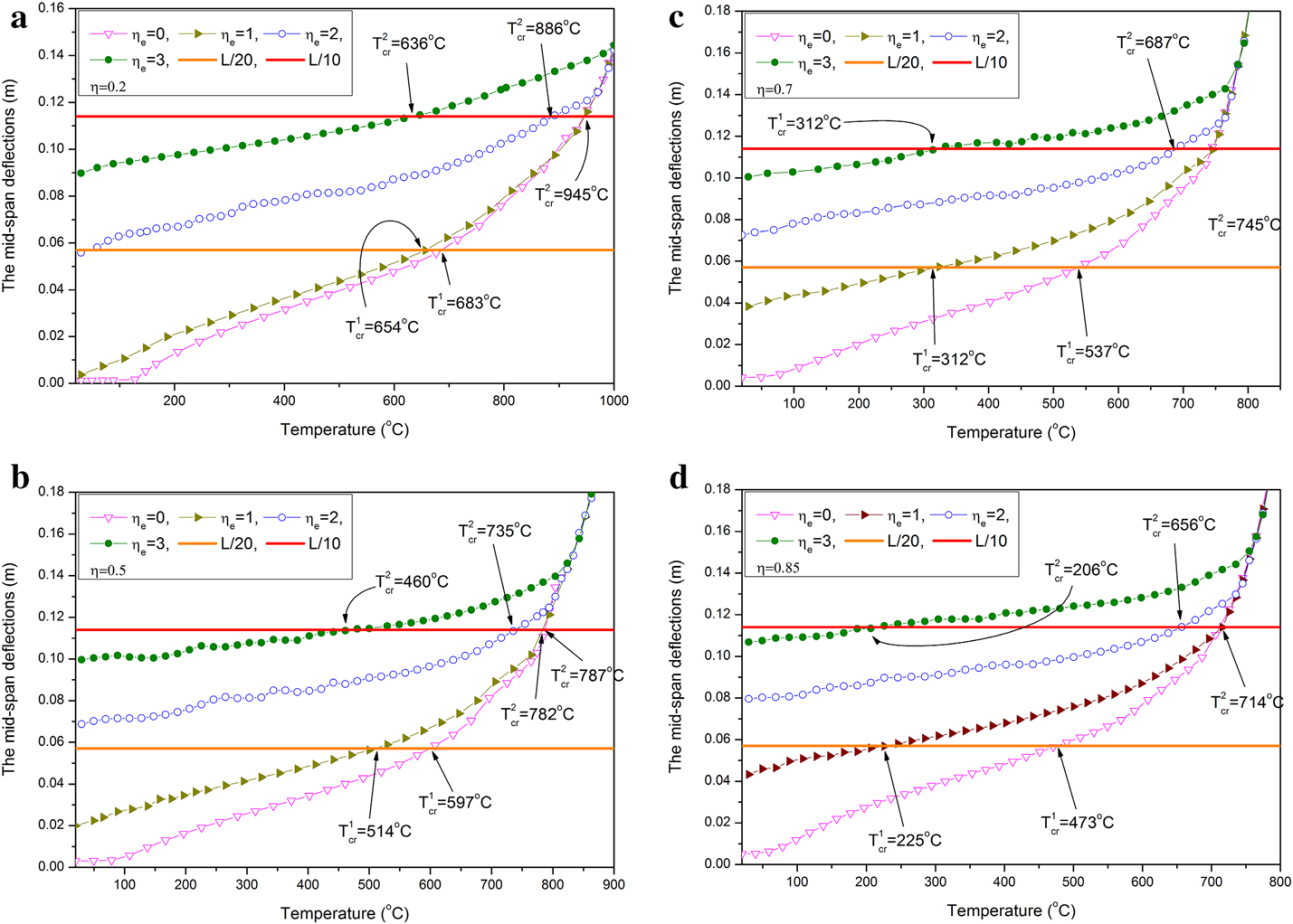
**a** Curves of the mid-span deflection against the temperature for load ratio $\eta = 0.2$. **b** Curves of the mid-span deflection against the temperature for load ratio $\eta = 0.5$. **c** Curves of the mid-span deflection against the temperature for $\eta = 0.7$. **d** Curves of the mid-span deflection against the temperature for $\eta = 0.85$

For the four cases of load ratio ($\eta = 0.2,0.5,0.7,0.85$), the curves of the mid-span permanent deflection against temperature are presented in Fig. 6a–d. The four curves of each figure correspond to the four cases of ELR $\eta_{e} = 0,1,2,3$, respectively, in which $\eta_{e} = 0$ denotes the absence of a blast load. By observing each curve of these figures, the deflections of the beam can be increased through blast loading. Moreover, if the increases in deflection are as large as the rise in ELR, then the limiting temperature of the beam will be affected.

To analyze variations in critical temperature, the L/20 and L/10 horizontal lines are also depicted in the figure. The maximum permanent deflections exceed the L/20 horizontal line for the two cases of ELR ($$\eta_{e} = 2,3$$). Therefore, only an L/10 horizontal line can be used to determine the critical temperature of cases with relatively high ELR. Obviously, a high ELR such as $$\eta_{e} > 2$$ cannot been applied if excessive deformations are not allowed in the fire-resistant design. Critical temperatures decrease as ELR increases, as shown in Fig. 6a. The critical temperatures are $$T_{cr}^{1} = 683$$ °C and $$T_{cr}^{1} = 654$$ °C, which correspond to $$\eta_{e} = 0$$ and $$\eta_{e} = 1$$, respectively. The difference between these values is only $$\Delta T_{cr}^{1} = 683 - 654 = 29$$ °C. However, this difference increases as load ratio $$\eta$$ increases, as shown in Fig. 6b–d. The difference in the critical temperature corresponding to $$\eta_{e} = 0$$ and $$\eta_{e} = 1$$ for $$\eta = 0.5,0.7,0.85$$ is $$\Delta T_{cr}^{1} = 597 - 514 = \,$$$$83\;{}^{\circ}{\text{C}}$$, $$\Delta T_{cr}^{1} = 537 - 312 =$$ 225 °C, and $$\Delta T_{cr}^{1} = 473 -$$$$225 = 248$$ °C, respectively.

The four curves on each figure tend to be superimposed as temperature increases, thus indicating that the influence of blast load has been exceeded by the serious deterioration of the strength and stiffness of the material caused by the elevated temperature. Thus, the deformation of the beam induced by the blast load no longer has a leading role. This effect is striking when the blast load is small, such as when $$\eta_{e} = 0$$ and $$\eta_{e} = 1$$, which correspond to the situation when the second critical temperatures $$T_{cr}^{2}$$ are the same. However, the influence of the blast load on displacement also increases and the second critical temperature decreases when ELR increases, such as when $$\eta = 0.2$$. The second critical temperatures corresponding to $$\eta_{e} = 2$$ and $$\eta_{e} = 3$$ are $$T_{cr}^{2} = 886$$ °C and $$T_{cr}^{2} = 636$$ °C, respectively. The difference is $$\Delta T_{cr}^{2} = 886 - 636 = 250$$ °C. When $$\eta = 0.5,0.7,0.85$$, the difference of the second temperature corresponding to $$\eta_{e} = 2$$ and $$\eta_{e} = 3$$ is $$\Delta T_{cr}^{2}$$$$= 735 - 460 = 275\,^{\circ}{\text{C}}$$, $$\Delta T_{cr}^{2} = 687 - 312 = 375$$ °C, and $$\Delta T_{cr}^{2} = 656 - 206 = 450$$ °C, respectively. Thus, the difference in critical temperature also increases as load ratio increases.

### Steel beam subjected to fire followed by an explosion

The hinged–hinged beam is subjected to the following loads in succession:

① First, a transverse dead load *P* is applied at the mid-span, $P = \eta P_{c}$.
② Next, the fire load is applied until a given temperature is reached.
③ An explosion load with a rectangular pulse is then applied. ELR and duration are $\eta_{e} = P_{d} /P_{c}$ and $t_{0} = 0.05$ s, respectively.

Obviously, the response of the steel beam under these combined loads is complicated, particularly because the blast or impact load is applied on the steel beam that is subjected to the fire load. The sensitivity of the strain rate of the materials is different from that when temperature is normal. Thus, both parameters *D* and *q* of the Cowper–Symonds equation are related to temperature. However, such test data are lacking. In a recent study on the behavior of a steel beam subjected to fire and subsequent impulsive loading (Xi et al. 2014), an assumption of the relationship between the parameters of strain rate and temperature was proposed. In the present study, we still use this assumption. Although this assumption is not necessarily consistent with reality, the difference should not be significant and the emphasis of the present study is to describe the method. In any case, the real structural behavior can be effectively simulated by using the proposed computing model if the test accurately provides the temperature-dependent strain rate parameters.

**Fig. 7:**
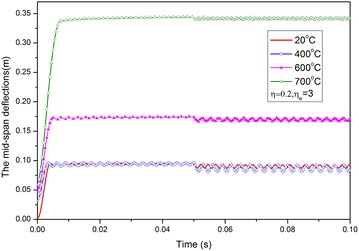
Curves of mid-span deflection against time

For load case $\eta = 0.2$, given temperatures at 20, 400, 600, and 700 °C, and ELR $\eta_{e} = 3$, the time history curves of the mid-span deflection are presented in Fig. 7, in which 20 °C corresponds to the case when a fire load is unavailable. An obvious increase in deflections occurs when the given temperature is greater than 400 °C. However, the difference between the deflections that correspond to 20 and 400 °C is minimal. When the given temperature is <400 °C, material stiffness is reduced and thermal expansion deformation is produced, whereas the strength of the material only deteriorates at temperatures higher than 400 °C. Therefore, for a beam under these loads, the main factor that generates a rapid increase in displacement is deteriorating strength caused by elevated temperatures, whereas the influences of thermal expansion deformation and stiffness deterioration caused by changes in temperature are minimal.

**Fig. 8:**
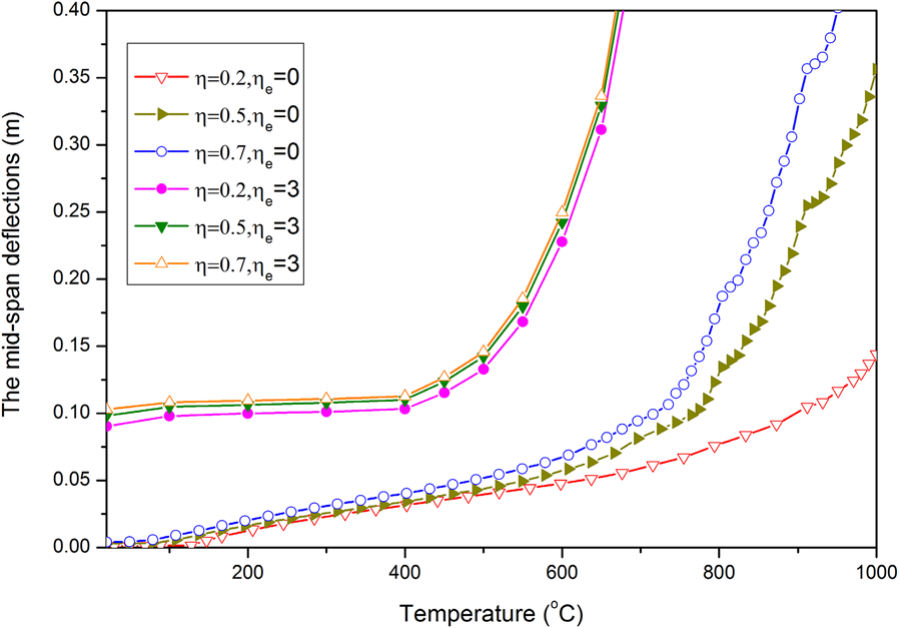
Curves of the mid-span deflection against temperature

Based on the curves of the deflection–time history shown in Fig. 7, the curves of the mid-span permanent deflection (regarded as the even deflection of residue elastic vibration) against temperature under several load ratio cases $\eta = 0.2,0.5,0.7$ and ELR $\eta_{e} = 3$ are presented in Fig. 8. For comparison, the curve corresponding to the case when $\eta_{e} = 0$ is also depicted in this figure. The shapes of the curves have obviously changed, and beam deformation increases as a result of the blast load. Thus, the critical temperature decreases. However, deflections do not always increase obviously when temperature increases, such as when *T* < 400 °C. This phenomenon can be attributed to the increase in the axial thermal expansion deformation of the beam as temperature increases to 400 °C, thus indicating that the deformation mechanism is dominated by axial thermal expansion. When temperature exceeds 400 °C, the strength of the material starts to deteriorate and the deformation mechanism assumes a leading role in bending deflection. Then, deflection rapidly increases as temperature continues to rise. Obviously, the failure limiting temperature can be easily determined for such deformation mode.

Compared with the three curves that correspond to the case with no blast load, the three curves of $\eta_{e} = 3$ are significantly closer to one another, thus implying that the influence of the first dead load with different load ratios on the deformation of the beam is minimal.

**Fig. 9:**
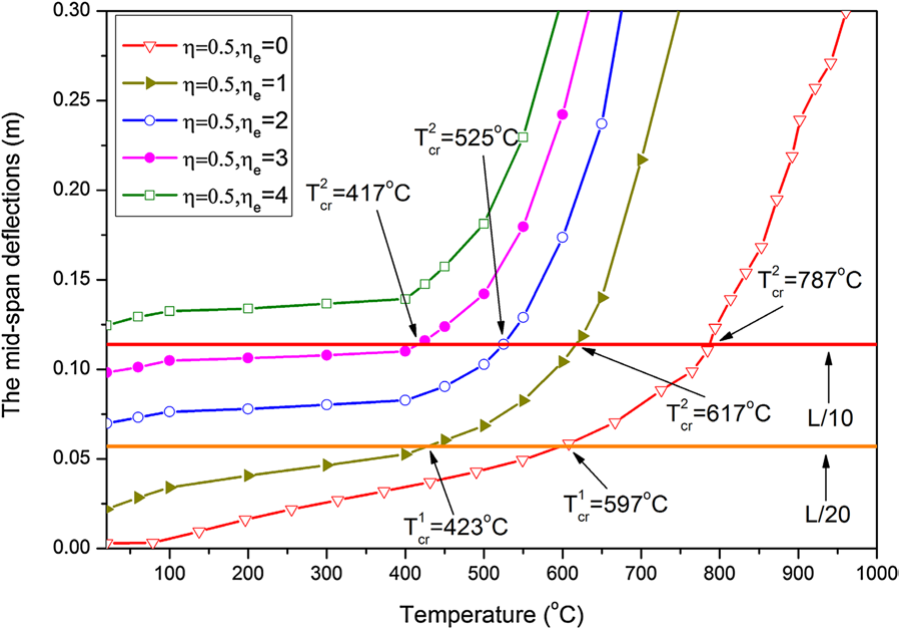
Curves of mid-span permanent deflections against temperature

For $\eta = 0.5$ and $\eta_{e} = 0,1,2,3,4$, the curves of the mid-span permanent deflection-temperature are presented in Fig. 9. Deflections increase as blast loads increase, and each curve exhibits characteristics that show that deflection increases dramatically as temperature rises. Thus, a failure temperature exists for each load case.

The L/20 and L/10 horizontal lines are also depicted in Fig. 9, such that the corresponding critical temperature can be easily determined. The results are presented in Table 2. For comparison, the critical temperatures obtained in phase 3.2 are also provided. Table 2 shows that the two results are different. The critical temperature of the steel beam that is subjected to fire followed by an explosion is lower than that of the beam that is subjected to explosion followed by fire. These results show that different loading sequences can lead to various response behaviors, thus affecting the critical temperature of a structure.

**Table 2:** Comparisons of critical temperatures

$\eta = 0.5$	$T_{cr}^{1}$ (°C)	$T_{cr}^{2}$ (°C)		
	$\eta_{e} = 1$	$\eta_{e} = 1$	$\eta_{e} = 2$	$\eta_{e} = 3$
Scenario (c)	514	782	735	460
Scenario (d)	498	664	574	504

**Fig. 10:**
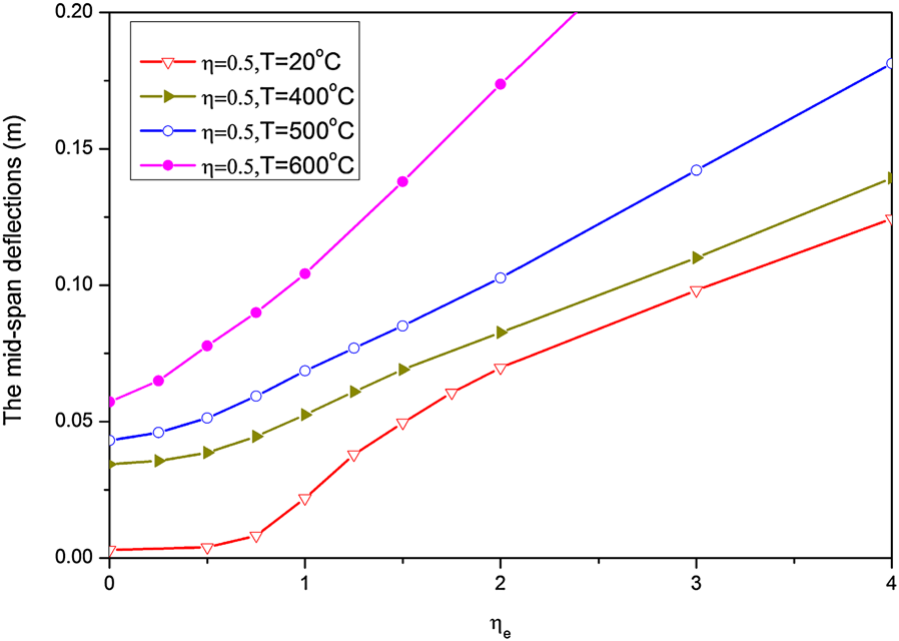
Curves of the permanent deflection against ELR

In Fig. 10, the curves of the mid-span permanent deflection against ELR are depicted for the cases of $\eta = 0.5$ and different temperatures. Obviously, displacement does not increase dramatically as ELR increases, which may be attributed to strain rate and inertia inhibiting the occurrence of a phenomenon similar to that of the static case. Thus, the corresponding dynamic limiting loads are not determined. Therefore, the explosion-limiting load will also not exist in this case when the failure of materials is not considered.

**Fig. 11:**
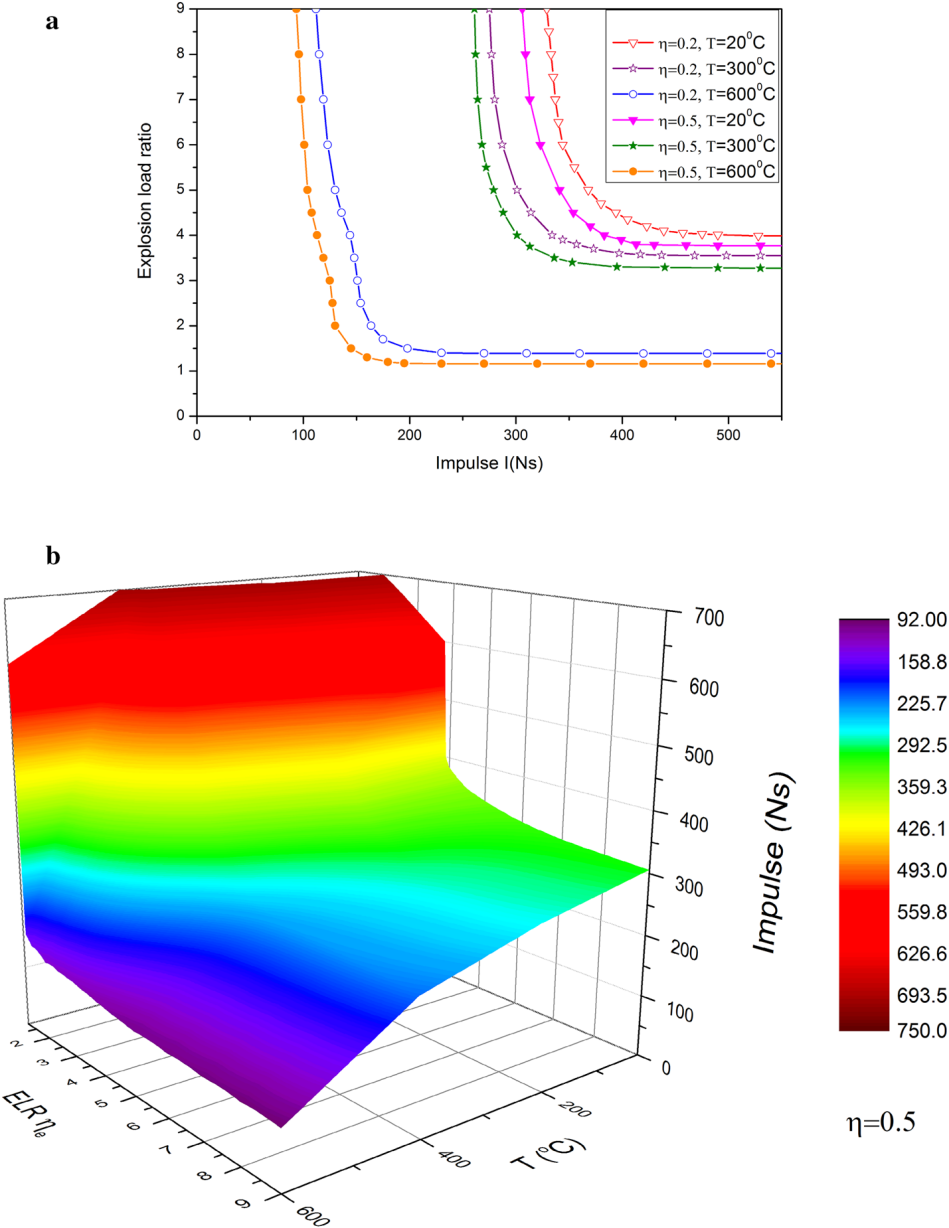
**a** The pressure–impulse diagram under three temperature conditions. **b** The pressure–temperature–impulse diagram for η = 0.5

To evaluate the effects of temperature on the pressure–impulse diagram, Fig. 11a represent several ELR–impulse curves in three temperature conditions and two statical load ratios. The area of the safety zone at the lower left of the curve decreases as temperature rises. In particular, the areas of zones with temperatures of 300 and 600 °C are significantly different, and thus, the safety zone is seriously reduced by high temperatures. By contrast, the influence of the statical load ratio is insignificant. For η = 0.5, an iso-damage surface on the three dimensional parametric space of temperature, ELR and impulse is presented in Fig. 11b, this 3D surface divides the space into two regions. The region below the surface is the safe spatial zone while the region above it is unsafe spatial zeon. When designing the structural fire and explosion resistance, it should be ensured that the temperature, ELR and impulse is located in the safe region.

## Conclusions

Based on the minimum principle of acceleration in the dynamics of elastic–plastic continua at finite deformation, an FD computing model to simulate the large deflection behavior of a steel beam that is subjected to the combined loads of static, fire, and explosion is presented. Response analysis for a hinged–hinged steel beam is conducted for several load cases by using the proposed model. The innovations of this study are listed as follows.

- The criteria that can determine the limiting temperature of a large deflection steel beam under fire are further confirmed, i.e., the first and second limiting temperatures that correspond to the bending and stretching plastic hinges, respectively. Both temperatures can be obtained from the curve of the dimensionless axial force against temperature when the dimensionless catenary force is zero and one, respectively.
  The numerical results also show that the limiting temperatures determined by the first criterion are closer to the critical temperature determined by the L/20 line, whereas the limiting temperatures determined by the second criterion are slightly higher than the critical temperature determined by the L/10 line.
- For beams subjected to explosion followed by fire, the blast load affects the limiting and critical temperatures. The critical temperatures are related to ELRs, and the difference of both critical temperatures corresponding to two low ELRs is related to the static load ratio. Such difference increases as $\eta$ increases.
  The influence of the blast load decreases as temperature rises, and the deformation induced by the blast loads no longer has a leading role. These effects are obvious in low ELRs, and the key factor that affects the critical temperature is static ratio.
- For beams subjected to fire followed by an explosion, the numerical results show that the influence of thermal expansion deformation and the reduction of stiffness caused by temperature change in the permanent deflection of the beam are minimal. Moreover, no serious deformation is observed within the scope of *T* < 400 °C. However, when temperature exceeds 400 °C, the influence of the reduction in material strength on permanent deflection is significant, and deflection rapidly increases as temperature rises. High temperatures also seriously influence the pressure–impulse diagram of a beam. An iso-damage surface on temperature, ELR and impulse space is introduced to distinguish safe and unsafe regions, which could be used in the structural design for the resistance of fire and blast loads.
- Two cases of explosion and fire loading sequences are compared. The displacement response and the critical temperatures are different for the two cases. The critical temperature of the beam that is subjected to fire followed by an explosion is lower than that of the beam that is subjected to an explosion followed by fire.
- Explosion limit load do not exist because the effects of strain and inertia when the material failed are not considered. The area of the safety zone in the pressure–impulse diagram decreases as temperature rises, whereas the influence of the static load ratio is insignificant.

In summary, a method for analyzing the large deflection behavior of restrained steel beams under the combined loads of explosion and fire is presented. The conclusions obtained through numerical results are conducive to further understanding on structural response and failure. Undoubtedly, this study is beneficial to designing fire- and explosion-resistant structures.
